# New Method of Treating Hooping Cough

**Published:** 1850-09

**Authors:** Eben. Watson


*New Method of Treating Hooping-Cough.*---By Dr. Eben. Watson.

[The author, before proceeding to mention his method of treating Hooping-Cough, alludes to the prevailing theories of the disease; he observes:]

There are four different views of the nature of the Hooping-Cough, each of which has had its advocates. According to one, it depends on inflammation of the brain and its membranes, from which the inflammation extends to the respiratory surfaces; while another class of pathologists maintain exactly the opposite—viz., that the inflammation originates in the bronchial membrane, and extends rapidly to the brain and medulla oblongata. A third theory is, that while the brain and medulla may be unaffected, the essence of the malady lies in a peculiar irritation of the trunks of the great nerves supplying the larynx, diaphragm, and stomach, viz., the pneumogastric and phrenic---without any bronchial inflammation necessarily existing at all. And the fourth theory is, that the poison of the Hooping-Cough first causes inflammation of the respiratory surfaces, and very speedily affects the nerves, so as to render the glottis peculiarly irritable.

This last-mentioned theory is substantially the one which I adopt, as being the simplest, and nearest to the truth. I believe that in a case of uncomplicated Hooping-Cough the morbid poison first inflames the mucous membrane lining the pharynx and the upper part of the larynx; that there the extreme branches of the sensory nerve, the superior laryngeal, become affected in some peculiar way which I shall not attempt to explain; and that, finally, the motor nerve, the recurrent laryngeal, is excited into reflex action. Hence the ordinary progress of the symptoms, which first resemble those of a common cold or slight bronchitis—differing, however, from the latter, in the absence of any constitutional or stethoscopic indications of inflamed bronchi, as well as in the periodic character of the cough. Along with this symptom may also be ranged certain peculiar pains of the throat and neck, which are often complained of, and seem to indicate the effect of the poison on the sensory nerve of these parts. Then follows the distinctive symptom of the affection---viz., the whoop, or drawback---which is produced by the excitement of the inferior laryngeal nerve and consequent spasm of the muscles of the glottis. Such are the symptoms which, in my opinion, are alone essential to Hooping-Cough. Few cases of that disease, however, proceed far on their course without another symptom---viz., vomiting, during, or towards the end of the paroxysms of coughing, which is doubtless caused by an extension of the morbid influence to the branches of the pneumogastric which supply the stomach.

[Viewing the disease, then, as essentially an inflammation of the pharyngo-laryngeal mucous membrane, with peculiar irritability of the glottis, it occurred to Dr. Watson, that an efficient remedy might be found in cauterization of the larynx, according to the plan of Dr Horace Green. (See H. Y. A., vol. vi., p. 186.) He accordingly put it in practice in the following cases.]

"The first case in which I attempted it was that of a weakly boy, about eight years old, in whom the disease was already at its height. He had a severe paroysm of coughing regularly every quarter of an hour; he was already much debilitated, and in constant danger of having some serious lesion produced in his delicate lungs. From the first time that I applied the solution to the glottis, the severity of the cough was mitigated, and, after repeating the application every second day for about a week, he was found not to whoop at all. The boy then made a speedy and complete recovery of his ordinary strength, under the use of the Cod-Liver Oil.

"The sister of this patient was also seriously affected by the same disease, and the application of a solution of Caustic was likewise used in her case. But its effects were not so striking as in the other case, owing to the presence of lobular Pneumonia of the posterior part of the left lung. Nevertheless, the paroxysms of the Hooping-Cough diminished in frequency and in violence under its use; and after the Pneumonia had been subdued by pretty active measures, the child ultimately made a good recovery.

"My third case was that of about six years of age, in whom the disease had nearly passed without much treatment of any kind having been employed; but he still was harrassed with frequent and pretty violent fits of coughing; and when excited or frightened, he still had that mode of drawing in his breath, popularly known as "the drawback." Two or three applications of the solution to the glottis and larynx of this child were sufficient to accomplish a perfect cure of his symptoms; and a short sojourn in the country, with the use of a tonic, completed his restoration to health and vigor."

Dr. Watson has since treated, in a similar manner, several other cases, the results of which are equally favorable with those already mentioned. None of his patients continued to whoop more than eight or ten days after the solution of Caustic began to be applied to the glottis; and from the first of such applications, the mitigation was very marked. In some cases of children living in the same house with patients affected with Hooping-Cough, and who exhibited symptoms of the commencement of that disease, such as frequent fits of coughing, especially during the night, and when suddenly agitated, with absence of co-existing Fever, and of the physical signs of Bronchitis, &c., he has, by applying the solution of Caustic to their larynges, succeeded in dissipating the symptoms alluded to, and restoring the children to health.

The strength of the solution is grs. xv. to the ounce, applied every second day by means of whalebone tipped with sponge, at first to the pharynx only. The mode of doing this is essentially the same as by Dr. Horace Green, and need not be repeated.]—*Ranking's Abstract.*

